# Identification of 12 cancer types through genome deep learning

**DOI:** 10.1038/s41598-019-53989-3

**Published:** 2019-11-21

**Authors:** Yingshuai Sun, Sitao Zhu, Kailong Ma, Weiqing Liu, Yao Yue, Gang Hu, Huifang Lu, Wenbin Chen

**Affiliations:** 1 BGI-Wuhan, BGI-Shenzhen, Wuhan, 430075 China; 2 China National GeneBank, BGI-Shenzhen, Shenzhen, 518116 China

**Keywords:** Cancer genomics, Computational science

## Abstract

Cancer is a major cause of death worldwide, and an early diagnosis is required for a favorable prognosis. Histological examination is the gold standard for cancer identification; however, large amount of inter-observer variability exists in histological diagnosis. Numerous studies have shown cancer genesis is accompanied by an accumulation of harmful mutations, potentiating the identification of cancer based on genomic information. We have proposed a method, GDL (genome deep learning), to study the relationship between genomic variations and traits based on deep neural networks. We analyzed 6,083 samples’ WES (Whole Exon Sequencing) mutations files from 12 cancer types obtained from the TCGA (The Cancer Genome Atlas) and 1,991 healthy samples’ WES data from the 1000 Genomes project. We constructed 12 specific models to distinguish between certain type of cancer and healthy tissues, a total-specific model that can identify healthy and cancer tissues, and a mixture model to distinguish between all 12 types of cancer based on GDL. We demonstrate that the accuracy of specific, mixture and total specific model are 97.47%, 70.08% and 94.70% for cancer identification. We developed an efficient method for the identification of cancer based on genomic information that offers a new direction for disease diagnosis.

## Introduction

Cancer is the most common risk that threatens human health worldwide. There are more than 100 types of cancer, including cancers of the breast, skin, lung, colon, prostate and ovaries. In the United States, 1,735,350 new cancer cases and 609,640 cancer deaths will be reported in 2018^1^. It is known that cancer is mainly caused by harmful mutations in proto-oncogenes, tumor suppressor genes and cell cycle regulator genes. Previous studies indicated that *p53* activates DNA repair proteins and inhibits the occurrence of various types of cancer^2^. In breast cancer, high penetrance mutations in *BRCA1* and *BRCA2* cause a loss of tumor suppressive function which correlates with an increased risk of breast cancer^3^. In addition, *C21orf58* and *ZNF526* also have functional roles in the control of breast cancer cell growth^4^. There are published reports that stomach cancer may be caused by the accumulation *PBLB2* and *ATM* mutations^5^. BLCA (Bladder Urothelial Carcinoma) is a major cancer of the urinary system. TCGA researchers have identified many mutated genes that are involved in the cell cycle, DNA repair and chromatin modifications in BLCA. *BLCA-4*^6^, a nuclear matrix protein, plays a major role in bladder cancer carcinogenesis. Although many genes that have been found have major roles in the occurrence and spread of cancer, the pathogenic mechanisms of gene mutations and interactions between genes are largely unknown. In this work we studied twelve cancer types including BLCA, BRCA (breast adenocarcinoma), COAD (colon adenocarcinoma), GBM (glioblastoma multiforme), KIRC (kidney renal clear cell carcinoma), LGG (low grade glioma), LUSC (lung squamous cell carcinoma), OV (ovarian carcinoma), PRAD (prostate adenocarcinoma), SKCM (skin cutaneous melanoma), THCA (thyroid carcinoma) and UCEC (uterine corpus endometrial carcinoma). With the development of DNA sequencing and bioinformatics analysis methods, we have been able to identify additional genomic mutations and have accumulated a large amount of data. Methods for identifying correlations between mass genomic variations and cancer are urgently required.

Deep learning methods, such as Alpha Go^7^ and object recognition^8^, exceed human performance in visual tasks and are flexible and powerful analytical techniques for dealing with complex problems. Deep learning is a high-level abstraction algorithm for solving classification and regression problems. Through deep learning and pattern mining of data, it identifies complex structures in massive data sets and has great potential for applications in genetics and genomics^9,10^. As a novel technique, a number of cases were shown to provide better performance in biological applications^11^. Deep learning methods can be used to learn how to recognize the locations of splice site promoters and enhancers^12^. Deep learning methods also have many applications in the prediction of protein secondary structure and function^13^. More accurate identification of phenotypes would improve study efficiency through a convolutional neural network^14^, which is one image recognition algorithm of Deep learning methods. Researchers also found that the skin cancer identification rate using deep neural networks was more accurate than that determined by dermatologists^15^. Kun-Hsing identified thousands of objective features from the images, built and evaluated machine learning classifiers to predict the survival outcomes of lung cancer patient^16^.A deep learning model using non-invasive CT images was used to predict EGFR mutation status for patients with lung adenocarcinoma^17^. Artificial intelligence algorithms could achieve higher tumor-level sensitivity than pathologists^18^. Automatic Detection of Cerebral Microbleeds From MR Images was identified by 3D Convolutional Neural Networks^19^. A deep convolutional neural network was constructed to distinguish pathologically confirmed Prostate cancer^20^. Deep learning methods use multiple layers of nonlinear processing units for feature extraction and transformation to find deep relationships between complex variations under supervised or unsupervised procedures^21^.

Biological traits are the result of interactions between gene sequences and gene interactions under certain environmental conditions. The deep learning model is suitable for studying the relationship between these factors and the phenotype. We constructed a model for the identification of cancer based on genomic variations that we call “genomic deep learning” (GDL). GDL studies the relationship between genomic variations and traits through deep learning of genomes. Even though GWAS is used to identify associations between single nucleotide variations and cancer^22^, GWAS is based on linkage analysis to find the diseased genes and requires more intimate segregate sites^22^. However, deep learning models can take entire genome variations into account without the influence of segregate sites. Neural network algorithms are inspired by biological neural networks. It is possible and feasible to build a deep neural network (DNN) model for the identification of cancer via massive variants.

In this work we constructed 14 models including 12 specific models, a total-specific model and a mixture model for cancer risk identification using a deep neural network (DNN) within a TensorFlow^23^ framework (https://github.com/Sunysh/Genome-Deep-Learning). We used an exponential decay method to optimize the learning rate, L2 regularization^24^ to minimize overfitting, and a sliding average model to increase the robustness of the model. For each specific model meant to identify a certain type of cancer, the detection accuracy, sensitivity and specificity are more than 97%, 98% and 97%, respectively. The mixture model, which is able to distinguish all 12 types of cancer, exhibited comparable performance. The total-specific and mixture models also demonstrated comparable performance. Using our model, cancerous tissue can be identified more conveniently and timely, thus providing an opportunity for earlier treatment. This approach to genome deep learning offers a new direction for disease diagnosis while providing a new method to predict traits based on genomic information.

## Methods

### Genome deep learning methodology

**Figure 1:**
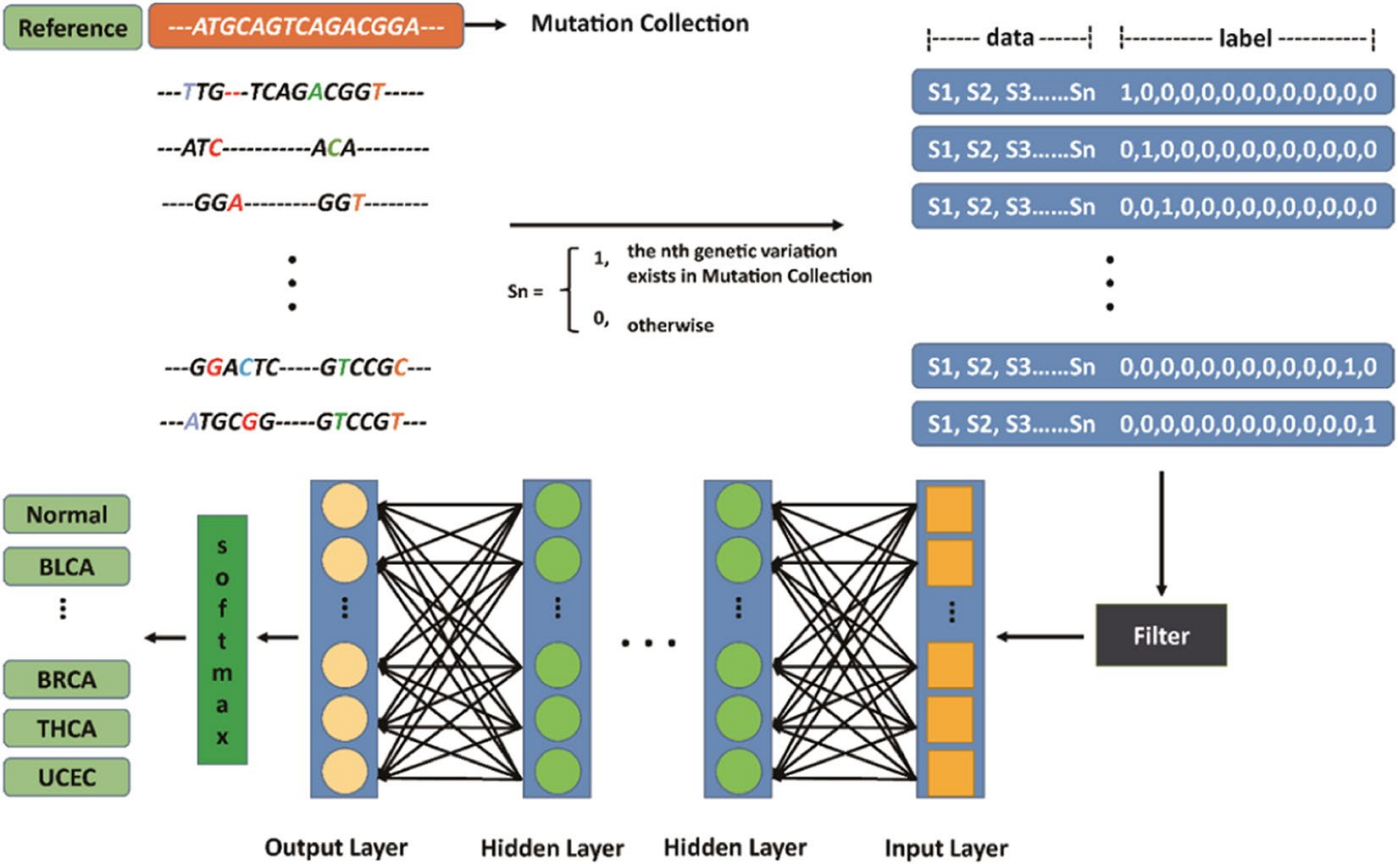
The architecture of genomic deep learning (GDL). The Mutation Collection used as reference. Point mutation transform the data and label through Sn rule.

Cancer is caused by the accumulation of harmful mutations^25^. Mutations occur all the time, especially during cell genome duplication, but most of the mutations are not on key genes. If the harmful mutations occur in the oncogene or tumor suppressor genes, the normal cells will become cancer cells. Changes in multiple genes are required to transform a normal cell into a cancer cell. To determine the relationship between mutations and cancers, we designed a deep learning method that we call genomic deep learning (GDL). GDL is a classification method for cancer identification. The architecture of our model contains feature selection, feature quantization, data filters and deep neural networks involving multiple hidden layers between input and output layers (Fig. 1).

GDL consists of data processing and model training. Data processing consists of three steps. First, the sequencing data are compared with a reference to obtain a point mutation file, and then the point mutation file is converted into a format of the model input. The third step is to filter the data after conversion formatting especially in specific model, because we only selected limited variation sites. In model training part, model was composed of four fully connected layers and softmax regression layer. ReLU (Rectified Linear Unit) was used as non-liner activation function in GDL model. L2 regularization was used to optimize model. The code that built model on github (https://github.com/Sunysh/Genome-Deep-Learning). Model training is DNN modeling and includes an input layer, multiple hidden layers, an output layer and a softmax layer. After training, a classification model is finally obtained.

### Model feature selection and quantification

To collect point mutations for the DNN model, we downloaded healthy tissues from the IGSR (The International Genome Sample Resource, http://www.internationalgenome.org/) and tumor tissues from the TCGA (https://portal.gdc.cancer.gov/). The WES tumor germline variants and somatic mutations were from twelve cancer types including BLCA, BRCA, COAD, GBM, KIRC, LGG, LUSC, OV, PRAD, SKCM, THCA and UCEC. Each type of tumor was comprised of 425, 1080, 493, 498, 376, 530, 561, 610, 503, 472, 504 and 561 samples for a total of 6083 datasets. We also downloaded blood WES sequencing data from 1,991 healthy individuals from the 1000 Genomes Project^26^ database. All selected dimension for model in Dataset1 and Dataset2 in Supplemental Dataset.

It is obviously impractical to select all of the point mutations as dimensions for the model because mass dimensions will increase the computation cost. To reduce the learning pressure brought about by highly redundant dimensions and to reduce the learning difficulty without affecting the accuracy of the model, we selected point mutations closely related to cancer from the TCGA as the dimension for the model. In specific models, we ranked the point mutations of each cancer according to the number of occurrences in this cancer group from high to low. We choose different ranked (1k, 2k, 3k, 4k, 5k, 6k, 7k, 8k, 9k and 10 K) points as the dimensions for model building. The results demonstrate that with an increase of the dimension, the accuracy will continue to improve (Supplementary Fig. 2). Finally, we chose 10,000 point mutations as the dimension for the specific models. The accumulation of harmful mutations is the root cause of cancer. In the development of cancer, the accumulation of mutations can be divided into two parts. The first part is the accumulation of mutations that occur that lead to the cancer, and the second part is the accumulation of mutations that occur after the cancer develops, which is the cause of tumor heterogeneity. Our goal was to determine the rules that gene mutations follow in converting healthy tissues to cancer, which is reflected in the effect of the mutations on the pathways involved. The difference in genetic mutations between patients with the same type of cancer is large because the effect on different pathways is similar. The second part of the mutation occurs on the basis of the first part of the variation. Using limited computing resources, we choose the position where the number of occurrences of the variation is more than 2 as the dimension in the total-specific model and the mixture model.

The Edico Genome Pipeline reduces the time required for analyzing an entire genome at 30x coverage from ~10 hours (BWA and GATK software)^27,28^ and was used to call variants for healthy tissues. The reference genome was GRCh38, which was downloaded from the National Cancer Institute website (https://gdc.cancer.gov/about-data/data-harmonization-and-generation/gdc-reference-files). Mutect2, a method that applies a Bayesian classifier to detect somatic mutations with very low allele fractions, requires only a few supporting reads, followed by carefully tuned filters to ensure that high specificity was used for calling cancer point mutations^29^.

In any application of deep learning methods, the researcher must decide which data format to provide as input to the algorithm. How to convert VCF data into GDL model input format becomes a significant challenge. To overcome this input format challenge, the HapMap^30^ project provided us with an approach. High risk sites were collected form the TCGA^31^ and we then sorted the collected sites by the frequency of occurrence in cancer patients named as Mutation Collection. Furthermore, variant sites from healthy people and cancer patients were assigned a score (“0” indicates different from Mutation Collection and “1” indicates the same as Mutation Collection) compared to the Mutation Collection. Finally, our input file became an array, for example: 1,1,0,0….1,0. As described above, our variant format was transformed into a different classifier, healthy or cancer. Each type of situation has its own classification label which is expressed by One-Hot Encoding. For example, the class labels “1,0,0,0,0,0,0,0,0,0,0,0” and “0,1,0,0,0,0,0,0,0,0,0,0” represent BLCA and BRCA, respectively. Other cancer types were treated as described above. Finally, the VCF files were transformed into two parts separated by a space. In part one, Sn had only two choices. A “1” indicates that the special individual genomic variation was the same as the Mutation Collection, and a “0” indicates that they were different. Sn represents each genomic variation in an n index. In part two, the class label indicates whether the individual was healthy or not (Fig. 1).

### Model function

The DNN model was composed of several computational layers. Each layer takes an input and produces an output, often computed as a non-linear function of weighted linear combinations of the input layer and adjusts each weight and threshold by accumulated error back propagation. In the forward propagation process, the output from each neuron is a nonlinear calculation of the weighted sum of the previous layer pointing to that neuron^32^. The formula used is

1 $$y=f(\mathop{\sum }\limits_{i=1}^{n}{w}_{i}{x}_{i}+b)$$

where y represents the output of activation, n represents the number of hidden units in the layer,*w*_*i*_ and *x*_*i*_ are the input of the activation, and b represents the bias terms.

Activation functions play an important role in deep learning because the combination of arbitrary linear models is still a linear model. To solve more complex problems, we used the activation function to achieve de-linearization. The commonly used activation functions are ReLU^33^, Sigmoid and Tanh^34^. The calculation using Sigmoid is relatively complex and requires a very long running time, and the gradient is easy to lose during the process of back propagation. Tanh also requires a large amount of calculation time. Although ReLU is relatively fragile, it requires a relatively small amount of computation, and it has faster convergence speed. The other advantage was that ReLU causes sparsity of the network and reduces interdependence of parameters that overcome the occurrence of overfitting problems. Formula 2 is the formula for the ReLU function:

2 $${f}_{RELU}(x)=\,\max (x,\,0)$$

After completing the current propagation, we use the loss function to represent the difference between the predicted and target values to evaluate the model’s effectiveness. The process of training the model is the process of decreasing the loss function. After the hidden layer of the model, the output of the hidden layer becomes a probability distribution through the softmax layer. We then use the cross entropy as a loss function to calculate the distance between the predicted probability distribution and the true probability distribution.

3 $${f}_{softmax}{(y)}_{i}=\frac{{e}^{yi}}{{\sum }_{i=1}^{n}{e}^{yi}}$$

4 $$H(y,y^{\prime} )=-\,{\sum }_{i}{y}_{i}^{^{\prime} }\,\log \,{y}_{i}$$

### Model optimization

To obtain a better model, we further optimized it based on back propagation^35^ and gradient descent. In the model training process, the learning rate controls the speed of the model update. If the learning rate is too high, the parameters will move back and forth on both sides of an acceptable value. On the contrary, if the learning rate is too small, convergence can be guaranteed, but the speed of optimization will be greatly reduced. Therefore, we used a more flexible learning rate setting method, i.e., exponential decay. With this method, a relatively large learning rate can be used to obtain a better result more quickly, and the learning rate is then gradually reduced with subsequent iterations, making the model more stable in the later period of training. Formula 5 is the formula for the exponential decay of the learning rate, where R represents the decayed learning rate, r represents the basic learning rate, d represents the decay rate, g represents the global step, and s represents decay step. Due to sequencing errors and the limitations of obtaining point mutation algorithms, false positive and false negative data are unavoidable in our data. If the model can remember the noise in each training data well, it will forget to learn the general trend in the training data. We use L2 regularization as an index of model complexity, and then add it to the loss function to reduce the model complexity and avoid overfitting problems. Formula 6 is the formula for the L2 regularization, where w_i_ represents weights. To improve the robustness of the model in the test data, we use the sliding average model which can reduce periodic interference and effectively remove the random fluctuations in the prediction. This approach maintains a shadow variable for each variable, and each time the variable is updated, the independent variable is also updated. Formula 7 is the formula for the shadow variable, where S represents the shadow variable, d represents decay and V represents a variable.

5 $$R=r\,\ast \,{d}^{\frac{g}{s}}$$

6 $$R(w)=\sum _{i=1}|{{w}_{i}}^{2}|$$

7 $$S=d\,\ast \,S+(1-d)\,\ast \,V$$

The DNN model was implemented in TensorFlow^23^ and the Google open source software library using data flow graphs and was trained on a Mac OS. In TensorFlow, networks are constructed and executed in a TensorFlow graph. Twelve cancer types, abbreviated as BRCA, OV, UCEC, LGG, LUSC, SKCM, GBM, LUAD, KIRC, THCA, PRAD and COAD were chosen to construct the DNN model.

### Model evaluation

Model evaluation produces an intuitive understanding of model reliability. To identify each cancer type, since it is a binary classification, we use accuracy, sensitivity and specificity to evaluate the classifiers’ performance. Since the total DNN model is a multi-class classification problem, we use accuracy, sensitivity and specificity to evaluate the total classifiers’ performance. Sensitivity, specificity and accuracy of the classification were calculated using results from all validation subsets. After the softmax function, if the probability score for a cancer was higher than the threshold value, the predictive diagnosis was a special cancer type.

$$\,\begin{array}{rcl}specificity & = & \frac{true\,negative}{negative}\\ sensitivity & = & \frac{true\,positive}{positive}\end{array}$$

## Results

### Cancer types and samples statistics

Genomic variation files for healthy people (1991) and cancer patients (6083) were obtained from the 1000 Genome Project Website and the TCGA online database. As shown in Table 1, the sample number of each tumor type ranges from 339 (KIRC) to 1,044 (BRCA). From 6,083 TCGA samples with available information, 71.61% (n = 4,356) were White, 9.73% (n = 592) were American, 3.98% were Asian and 14.68% have no race information. Patients (n = 6,083) were diagnosed between 55 and 74 years of age. The sex distribution had no serious effect, except for prostate adenocarcinomas (PRAD), which were all male, and ovarian carcinomas (OV) which were all female. Cancer staging plays an important role in determining treatment options. According to the cancer stage standards, all cancers were divided into four stages and one unidentified stage. From Table 1, we can see that GBM, LGG, OV, PRAD and UCEC have no clear stage. Sequencing reads of 1991 samples from the 1000 Genome project were analyzed using the Edico genome pipeline. We obtained 25 Tb of next-generation sequencing (NGS) data from the mainstream sequencing platforms including Illumine and Solid. The sequencing depth for healthy people ranged from 4 to 10 (Supplementary Table 1).

**Table 1:** Summary information of datasets from the TCGA and the 1000 Genome Project that were used in this study.

Cancer type	Samples	SNVs files	Age	Gender		Race				Tumor stage					Vital Status		
	(N)	(N)	(mean ± s.d.)	Male	Female	White	American	Asian	NA	I	II	III	IV	NA	Alive	Deceased	NA
				(%)	(%)	(N)	(N)	(N)	(N)	(N)	(N)	(N)	(N)	(N)	(N)	(N)	(N)
BLCA	412	425	73.1 ± 10.5	73.79	26.21	327	23	44	18	2	131	141	136	2	230	182	0
BRCA	1044	1080	67.0 ± 13.1	1.05	98.95	719	180	59	86	173	588	241	20	22	898	146	0
COAD	433	493	74.5 ± 13.6	51.97	48.03	212	59	11	151	90	166	118	46	13	332	99	2
GBM	396	498	63.1 ± 13.2	63.36	36.64	337	41	6	12	0	0	0	0	396	88	303	5
KIRC	339	376	69.1 ± 12.0	64.60	35.40	275	52	6	6	193	33	2	69	42	258	81	0
LGG	513	530	49.6 ± 12.8	55.27	44.73	472	22	8	11	0	0	0	0	513	386	126	1
LUSC	497	561	73.4 ± 9.1	73.84	26.16	348	30	9	110	242	160	84	7	4	279	218	0
OV	443	610	66.6 ± 11.7	0	100	376	31	14	22	0	0	0	0	443	188	253	2
PRAD	498	503	69.0 ± 7.1	100	0	147	7	2	342	0	0	0	0	498	488	10	0
SKCM	470	472	66.1 ± 14.9	61.70	38.30	447	1	12	10	77	140	185	23	45	249	221	0
THCA	496	504	55.5 ± 15.5	26.41	73.59	325	27	51	93	331	51	110	2	2	482	14	0
UCEC	542	561	72.2 ± 11.2	0	100	371	119	20	32	0	0	0	0	542	451	91	0
IGSR	1991	1991		∼	∼	∼	∼	∼	∼	∼	∼	∼	∼	∼	∼	∼	∼

### Accuracy of cancer identification

In specific model, 80% specific cancer samples used as training dataset and 20% used as testing dataset. The specific model took 10 K (Supplemental Table S6) variant sites’ transformation as input. After an extended period of data preparation and model training, an acceptable classification result was obtained. All specific models showed accuracy ranges from 97.47% (PRAD) to 100% (KIRC, LUSC, OV). The mean and standard deviation of accuracy are 98.70% and 0.91% respectively. The sensitivity of all specific model ranges from 95.79% (PRAD) to 100% (KIRC, LUSC, OV). The mean and standard deviation of sensitivity are 98.36% and 1.34%. The specificity ranges from 98.00%(UCEC) to 100% (KIRC, LUSC, OV). The mean and standard deviation of specificity are 99.03% and 0.7404% respectively.

**Figure 2:**
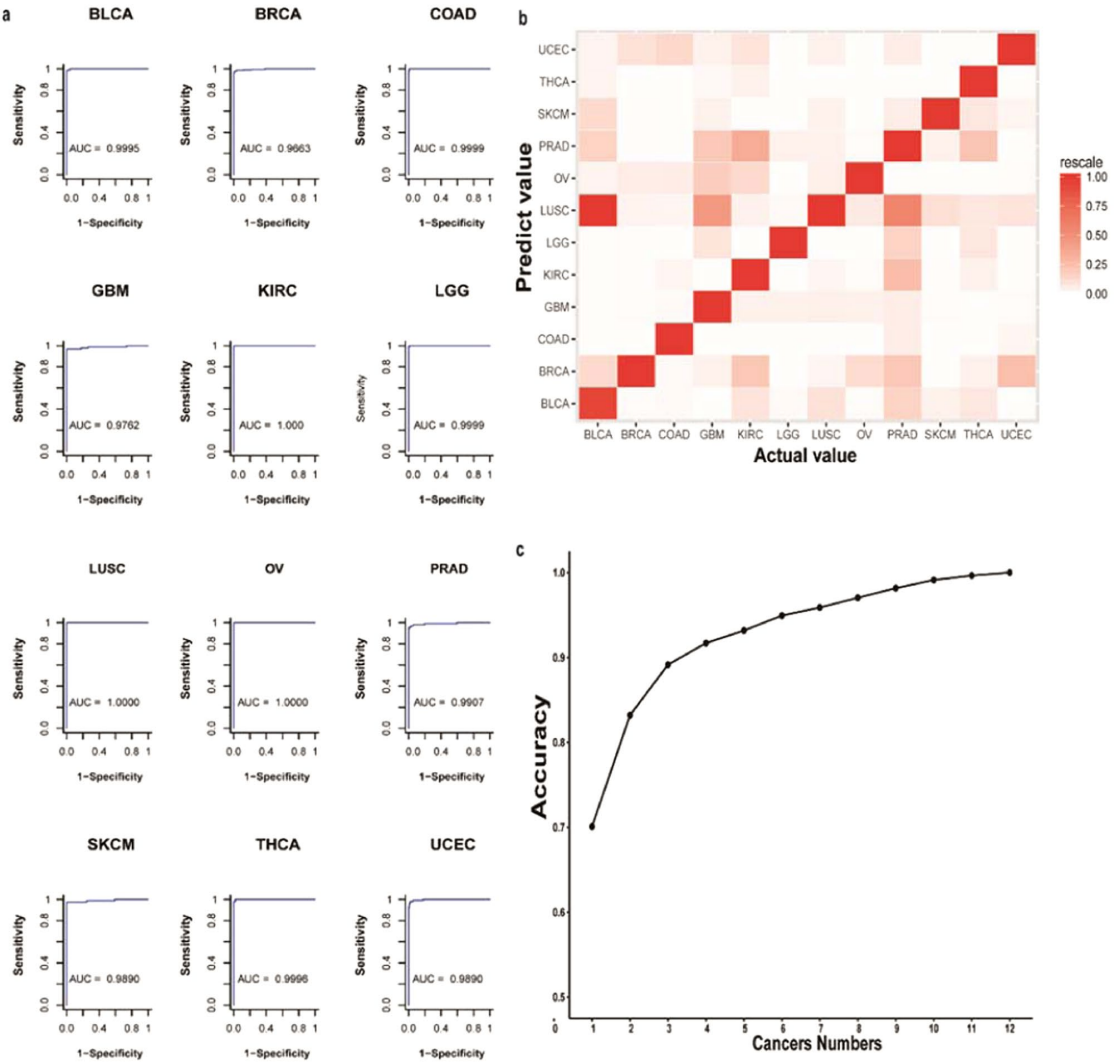
Cancer identification performance of 12 specific models and the mixture model. (**a**) The classification performance of 12 specific models. Using different thresholds, the sensitivity is the abscissa and the specificity is the ordinate, resulting in 12 ROC curves. The 12 ROC curves produce perfect classification results, and the area under the ROC curve (AUC) is greater than 96%. (**b**) Confusion matrix of the mixture mode. The abscissa indicates the label, and the ordinate indicates the predicted cancer type. LUSC is more obvious in the predictions, especially in the BLCA predictions, suggesting that many cancers are easily confused with LUSC. Cancers that are easily confused in model predictions may be similar in their genetic variations. (**c**) The accuracy of top-N at different forecasted quantities. The abscissa indicates different prediction numbers, and the ordinate indicates accuracy. The accuracy of the prediction result is 70.08%, and the accuracy of two prediction results is 83.20%, which provides support for the practical application of the model. The abscissa indicates the label, and the ordinate indicates the predicted cancer type.

In total-specific model, Model randomly selected 80% of 6803 samples (cancer) and 80% of 1991 samples (health) as training dataset. The rest dataset used as testing dataset. The accuracy, sensitivity and specificity of the total-specific model were 94.70%, 97.30% and 85.54%, respectively. We used ROC and AUC to evaluate the direct performance of the specific models (Fig. 2a). Each model exhibited a high AUC and was completely correct in four models, i.e., BLCA, KIRC, OV and THCA. Such high quality classification models demonstrate that significant differences exist between patients and healthy people (Supplementary Figs. 4–6). In mixture model, 80% of each cancer samples used as training dataset and 20% used as testing dataset. The accuracy, sensitivity and specificity of mixture model were 70.4%, 65.92%,96.27%.

To evaluate the model in a different aspect, we validated the DNN model using a four-cancer classification of cancer types according to the criteria staging system (tumor, node and metastasis, TNM) described in the AJCC Cancer Staging Manual^34^. The stage of the cancer is a key factor for determining the prognosis and will assist the doctor in determining the appropriate treatment. According to the criterion described in AJCC Cancer Staging Manual^34^, cancers can be divided into five levels based on the degree of tumor differentiation. In the first level (I level), the tumor has low pathogenicity and only occurs in specific areas such that the tumor has a better chance of being cured. In the fourth level (IV level), the tumor has a high degree of malignancy and has spread to other organs such that the tumor has a low probability of being cured. The last level does not meet the cancer staging standards described in the AJCC and is labeled as “None” because it is difficult to distinguish using the TCGA. For training models that use the cancer stage database, the mean accuracy for the DNN model is 97%, and the mean sensitivity and specificity is 98% and 97%, respectively (Table 2). Finally, for the mixture model, we used the data from each cancer type class to validate the DNN model.

**Table 2:** Summary of the GDL model classification performances.

Cancer type	Raw data		Filter						Accuracy (%)	Sensitivity (%)	Specificity (%)
			ALL		Train Data		Test Data				
	Cancer	Health	Cancer	Health	Cancer	Health	Cancer	Health			
BLCA	425	1991	417	216	341	165	76	51	98.43	98.68	98.04
BRCA	1080	1991	1073	586	856	471	217	115	98.19	97.70	99.13
COAD	493	1991	482	842	385	675	97	167	99.24	98.97	99.40
GBM	498	1991	478	435	385	345	93	90	97.81	96.77	98.89
KIRC	376	1991	372	189	288	161	84	28	100.00	100.00	100.00
LGG	530	1991	518	491	410	397	108	94	99.01	99.07	98.94
LUSC	561	1991	545	166	436	133	109	33	100.00	100.00	100.00
OV	610	1991	600	176	481	140	119	36	100.00	100.00	100.00
PRAD	503	1991	494	497	399	394	95	103	97.47	95.79	99.03
SKCM	472	1991	434	409	358	316	76	93	98.22	97.37	98.92
THCA	504	1991	503	241	405	190	98	51	97.99	97.96	98.04
UCEC	561	1991	549	1217	446	967	103	250	98.02	98.06	98.00
TOTAL	6613	1991	5733	1629	4585	1304	1148	325	94.70	97.30	85.54

To avoid the limitation of the specific model, we constructed a mixture model to distinguish all 12 types of cancer. The model is able to predict cancer with an accuracy of 70.08%, which is lower than that for the specific model. The accuracy of the mixture model is lower than with the specific cancer model, which is acceptable because it is a different cancer, and there is a great deal of similarity at the molecular level, causing the classification to be inaccurate^31^. Within the 12 cancers, the statistics suggested that the difference in the frequency of base mutations between different cancers is not very large. It was further demonstrated that although cancer tissues vary in form, there are large common genomic variations at the molecular level that lead to lower accuracy in the mixture model than in the specific model. Furthermore, the selection of reference sites is based on the frequency of sites in the cancer population. A high frequency of reference sites could promote better accuracy for multiply classifications in the mixture model.

**Figure 3:**
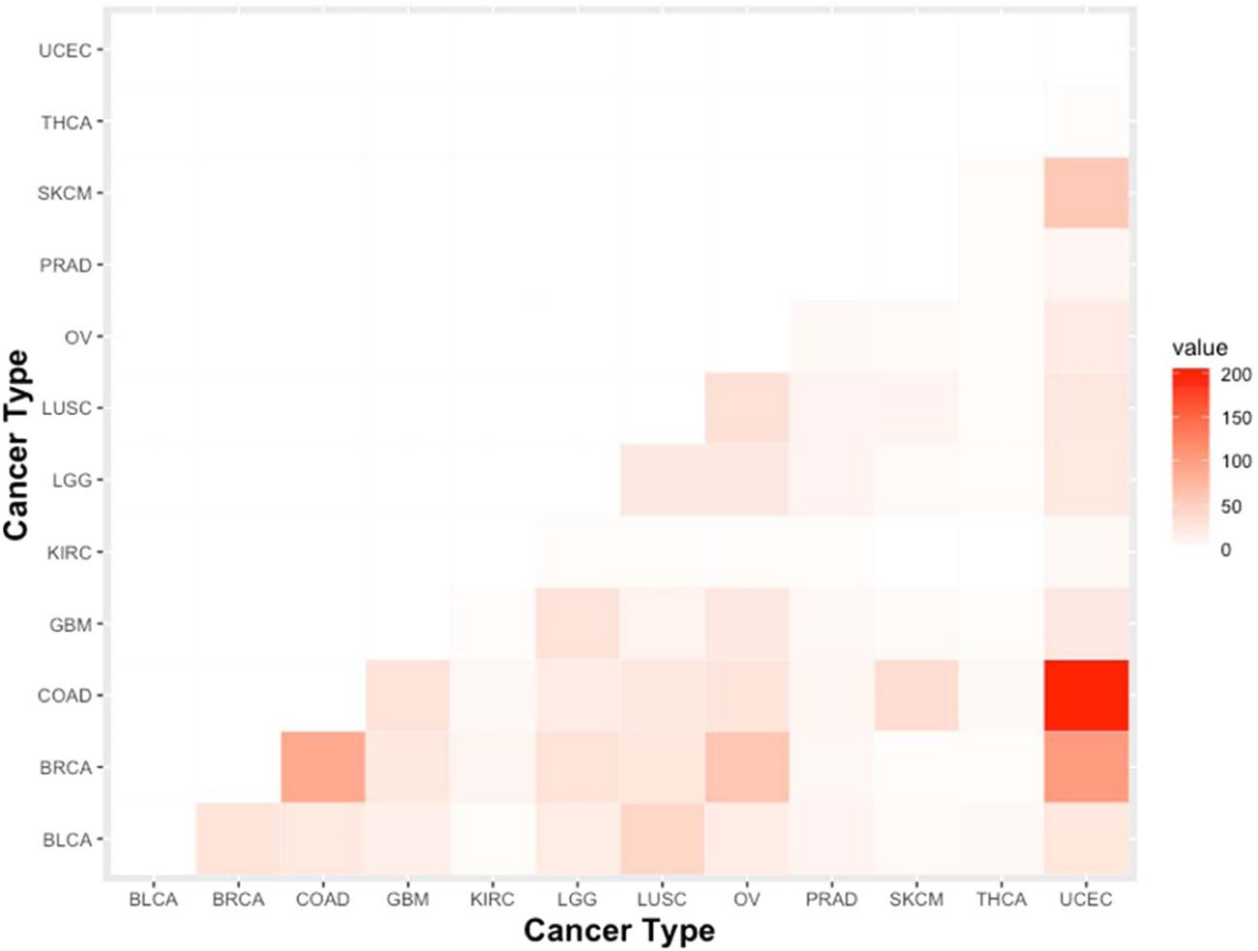
Mixed matrices of the same dimensions for different cancers. UCEC and COAD share the largest number of variant sites, followed by UCEC and BRCA. BRCA and COAD are relatively more common than other types of cancer.

To confirm a correlation between the number of common dimensions between different cancers and the judgment error in the mixed model we performed statistical analyses (Fig. 2b, Supplementary Figs. 3 and 5). In Fig. 3, we can see that the common dimension between UCEC and COAD is much larger than that between other cancer types. The common dimension of other groups (UCEC and BRCA, BRCA and COAD) is also higher than that between other cancer types, but much smaller than the common dimension between UCEC and COAD. However, as can be seen in Figs. 2 and 3, the ratio of false judgments between UCEC and COAD is lower than that between UCEC and BRCA, which indicates that the common dimensions have no correlation with model false judgments. The ratio of false judgments between BRCA and COAD is much lower than that between the two cancers in the common dimensions.

## Discussion and Conclusions

Our work provided a new method GDL (genome deep learning) involving DNN model for cancer identification based on genomic variation. GDL introduces a new method to identify cancer risk before cancer is diagnosed, which leave enough time for treatment. In this work we constructed 12 specific, a total-specific and mixture cancer identification models using a deep neural network (DNN) within a TensorFlow framework. All specific models showed accuracy ranges from 97.47% (PRAD) to 100% (KIRC, LUSC, OV). The accuracy, sensitivity and specificity of the total-specific model were 94.70%, 97.30% and 85.54%, respectively.

Comparing to traditional cytological identification of cancer, GDL is superior in at least two aspects. First, GDL method won’t be influenced by diagnostic instruments. With the implementation of large genome sequencing projects, more and more cancer associated variations especially the ones at low frequency will be identified so that our model will evolve rapidly and become more and more powerful. Second, our models are shown insensitive to cancer stage, making us able to confidently identify cancer at its early stage thus make time for treatment. Most importantly, machine learning is most effective in analysis of large, complex genomics data.

Non-invasive diagnostic methods such as liquid biopsy^36,37^ and non-invasive prenatal testing^38,39^ (NIPT) are growing rapidly and becoming more and more practicable. In liquid biopsy, cyclic tumor cells (CTCs) and circulating tumor DNA (ctDNA) fragments were collected from blood directly^40^, instead of invasive surgeries. Conjoining with genome sequencing, we believe our method could empower those technologies in accurately monitoring cancer risk in time.

Our models are still facing some limitations. For instance, more factors in addition to genomic variations (such as age, sex, transcriptome and proteome data) might be integrated into the model to promote prediction accuracy. Additionally, our models need to support more types of cancer to be distinguished. Those issues shall be resolved in the future. With the development of biology and deep learning, mass high reliability variants and algorithm will create a better model for cancer risk identification.

## Supplementary information

Supplemental table

Supplemental figure

Dataset1

Dataset2

## Data Availability

The 1991 normal sequencing data were download from The International Genome Sample Resource (ftp://ftp-trace.ncbi.nih.gov/1000genomes/ftp). The 6083 samples’ cancer data were download from TCGA (https://portal.gdc.cancer.gov/). The transformed data is available in GitHub (https://github.com/Sunysh/Genome-Deep-Learning).
