# Supplementary material for: Identification of 12 cancer types through genome deep learning
Source: Sci Rep. 2019 Nov 21;9:17256. doi: 10.1038/s41598-019-53989-3 (PMC6872744; doi:10.1038/s41598-019-53989-3)
Supplement: Supplementary file 1 — Supplemental table [file 41598_2019_53989_MOESM1_ESM.pdf]

Identification of 12 cancer types through genome deep learning

Yingshuai Sun[1,*], Sitao Zhu[1,*], Kailong Ma[2], Weiqing Liu[1], Yao Yue[1], Gang Hu[1], Huifang Lu[2], Wenbin Chen[2,#]

Supplementary Information

Table S1: Distribution of point mutation site

Variant types	Number
missense_variant.stat	10946
synonymous_variant.stat	4500
3_prime_UTR_variant.stat	3018
frameshift_variant.stat	2829
stop_gained.stat	1389
intron_variant.stat	1070
non_coding_transcript_exon_variant.stat	550
5_prime_UTR_variant.stat	543
downstream_gene_variant.stat	306
splice_region_variant.stat	195
upstream_gene_variant.stat	170
splice_acceptor_variant.stat	49
splice_donor_variant.stat	44
inframe_insertion.stat	30
inframe_deletion.stat	25
protein_altering_variant.stat	17
start_lost.stat	12
mature_miRNA_variant.stat	9
stop_retained_variant.stat	5
coding_sequence_variant.stat	3
stop_lost.stat	1

Table S2: Modeling accuracy of 12 kinds of cancer in different dimensions

**Cancer \ Dimension**	**1k**	**2k**	**3k**	**4k**	**5k**	**6k**	**7k**	**8k**	**9k**	**10k**
**BLCA**	0.9375	0.9438	0.9579	0.9604	0.9346	0.9636	0.9561	0.9487	0.9590	0.9843
**BRCA**	0.9132	0.9302	0.9419	0.9612	0.9496	0.9612	0.9671	0.9613	0.9600	0.9789
**COAD**	0.9811	0.9841	0.9718	0.9813	0.9831	0.9648	0.9954	0.9866	0.9922	0.9924
**GBM**	0.8298	0.8796	0.8917	0.9048	0.9412	0.9257	0.9487	0.9699	0.9657	0.9836
**KIRC**	0.8971	0.9487	0.9518	0.9884	0.9890	0.9560	0.9600	0.9811	1.0000	1.0000
**LGG**	0.8750	0.9569	0.9542	0.9930	0.9545	0.9699	0.9829	0.9891	0.9740	0.9950
**LUSC**	0.9223	0.9554	0.9661	0.9675	0.9766	0.9771	0.9852	0.9926	0.9928	1.0000
**OV**	0.9565	0.9590	0.9764	0.9771	0.9778	1.0000	0.9860	0.9932	0.9799	1.0000
**PRAD**	0.8228	0.8774	0.8833	0.9542	0.9313	0.9494	0.9222	0.9494	0.9626	0.9697
**SKCM**	0.9663	0.9888	0.9908	0.9750	0.9924	0.9781	0.9861	0.9671	0.9565	0.9882
**THCA**	0.9028	0.9278	0.9545	0.9583	0.9603	0.9695	0.9779	0.9784	0.9930	0.9866
**UCEC**	0.9650	0.9740	0.9682	0.9677	0.9549	0.9514	0.9671	0.9721	0.9527	0.9773

Table S3: Confusion matrix of mixture mode

**Label \ Predicate**	**BLCA**	**BRCA**	**COAD**	**GBM**	**KIRC**	**LGG**	**LUSC**	**OV**	**PRAD**	**SKCM**	**THCA**	**UCEC**
**BLCA**	29	4	0	0	0	0	31	1	5	4	1	1
**BRCA**	2	183	0	2	1	1	6	10	1	1	0	18
**COAD**	1	1	72	1	2	0	2	4	0	0	0	10
**GBM**	0	2	0	48	0	4	22	9	10	2	1	2
**KIRC**	2	5	0	1	23	0	1	3	8	0	1	2
**LGG**	1	0	0	3	0	70	1	0	3	0	0	0
**LUSC**	8	2	0	4	2	0	81	4	3	3	0	3
**OV**	1	11	0	4	1	0	6	90	1	0	0	0
**PRAD**	3	4	1	1	5	3	11	0	19	1	0	1
**SKCM**	3	0	0	1	1	0	9	0	3	82	0	0
**THCA**	2	1	0	0	1	2	2	0	6	2	26	0
**UCEC**	0	20	2	1	0	0	7	1	0	2	0	78

Table S4: Confusion matrix of mixture model two-way judgment error

Label \ Predicate	BLCA	BRCA	COAD	GBM	KIRC	LGG	LUSC	OV	PRAD	SKCM	THCA	UCEC
**BLCA**	29	0	0	0	0	0	0	0	0	0	0	0
**BRCA**	6	183	0	0	0	0	0	0	0	0	0	0
**COAD**	1	1	72	0	0	0	0	0	0	0	0	0
**GBM**	0	4	1	48	0	0	0	0	0	0	0	0
**KIRC**	2	6	2	1	23	0	0	0	0	0	0	0
**LGG**	1	1	0	7	0	70	0	0	0	0	0	0
**LUSC**	39	8	2	26	3	1	81	0	0	0	0	0
**OV**	2	21	4	13	4	0	10	90	0	0	0	0
**PRAD**	8	5	1	11	13	6	14	1	19	0	0	0
**SKCM**	7	1	0	3	1	0	12	0	4	82	0	0
**THCA**	3	1	0	1	2	2	2	0	6	2	26	0
**UCEC**	1	38	12	3	2	0	10	1	1	2	0	78

Table S5: Mixed matrices of the same dimensions for different cancers

Lable \ Label	BLCA	BRCA	COAD	GBM	KIRC	LGG	LUSC	OV	PRAD	SKCM	THCA	UCEC
**BLCA**	1369											
**BRCA**	30	994										
**COAD**	23	88	8497									
**GBM**	16	25	28	533								
**KIRC**	5	11	7	4	67							
**LGG**	19	30	20	29	4	216						
**LUSC**	43	26	25	14	3	24	889					
**OV**	19	60	27	24	3	24	32	471				
**PRAD**	13	10	11	10	4	13	12	7	102			
**SKCM**	6	4	36	6	0	6	12	6	1	6000		
**THCA**	8	2	7	3	1	4	5	2	5	5	98	
**UCEC**	26	103	899	24	8	23	25	21	11	56	3	8000
